# Surgical management of peripheral nerve symptoms following knee arthroplasty

**DOI:** 10.1186/s42836-025-00315-0

**Published:** 2025-06-06

**Authors:** Otis C. van Varsseveld, Floris V. Raasveld, Wen-Chih Liu, Justin McCarty, Caroline A. Hundepool, J. Michiel Zuidam, Ian L. Valerio, Kyle R. Eberlin

**Affiliations:** 1 Hand and Arm Center, Department of Orthopaedic Surgery, Massachusetts General Hospital, Harvard Medical School, Boston, MA 02115 USA; 2 Department of Surgery, University Medical Center Groningen, University of Groningen, Groningen, 9700 AB the Netherlands; 3 Division of Plastic and Reconstructive Surgery, Massachusetts General Hospital, Harvard Medical School, Boston, MA 02114 USA; 4 Department of Plastic, Reconstructive and Hand Surgery, Erasmus Medical Center, Erasmus University, Rotterdam, 3062 PA the Netherlands; 5 Department of Orthopaedic Surgery, Kaohsiung Medical University Hospital, Kaohsiung Medical University, Kaohsiung, Taiwan 80708 China

**Keywords:** Knee replacement, Chronic pain, Neuropathy, Peripheral nerve surgery

## Abstract

**Background:**

Neuropathic pain, weakness, and/or numbness can complicate partial or total knee arthroplasty (KA). This study evaluates peripheral nerve surgery following KA and proposes a treatment algorithm.

**Methods:**

Patients who underwent peripheral nerve surgery for neuropathic symptoms (neuropathic pain and/or motor dysfunction) following KA between 2012–2024 (≥ 3-month follow-up) were included. Demographics, comorbidities, and type of treatment were collected, and a cross-sectional survey assessed satisfaction (Patient Global Impression of Change, PGIC) and quality of life (EuroQol-5-Dimension-5-Level, EQ-5D-5L).

**Results:**

Twenty-seven lower extremities treated in 26 patients with a median age of 67.0 years (IQR: 58.0–71.8) were included. Surgical indications included neuropathic pain (*n* = 24/27, 88.9%), foot drop (*n* = 1/27, 3.7%), or both (*n* = 2/27, 7.4%). Median time between KA and nerve surgery was 29.5 months (IQR: 12.5–71.0). Procedures included saphenous or infrapatellar branch neurectomy with active management of the nerve ending (targeted muscle reinnervation (TMR) or regenerative peripheral nerve interface (RPNI)) (48.1%, *n* = 13), nerve decompression (40.7%, *n* = 11), or a combination of the two (11.1%, *n* = 3). Twenty-one patients (80.8%, 22 extremities) completed the survey with a median follow-up of 1.9 years (IQR: 1.1–4.2). Improvement (PGIC) was reported in 21 extremities (95.5%), the mean EQ-5D-5L index was 0.854 (± 0.102) (US general population: 0.851 (± 0.205)).

**Conclusion:**

Peripheral nerve surgery is beneficial for patients with neuropathic pain, numbness, and/or weakness following KA. We recommend common peroneal nerve decompression for lateral knee pain and/or foot drop, active saphenous nerve management with TMR or RPNI for medial knee pain, or a combination of the two based on the clinical scenario. These findings may aid in the decision-making process for patients with neuropathic pain following KA and warrant further validation in larger, prospective studies.

**Supplementary Information:**

The online version contains supplementary material available at 10.1186/s42836-025-00315-0.

## Background

Over 650,000 total and partial Knee arthroplasty (KA) surgeries are projected to be performed in the United States in 2025 for end-stage osteoarthritis, rheumatoid arthritis, and other knee pathologies [1]. In the total KA population, approximately 6% of patients report neuropathic pain. However, among patients who develop chronic post-surgical pain at 3 months after TKA, the overall prevalence of neuropathic pain is much higher, affecting 53–74%. Peroneal nerve palsy affects approximately 0.4% of all TKA patients, with higher rates (1.9%) observed specifically in patients with valgus deformities [2–6]. Since pain relief is a primary expectation of KA, persistent pain often leads to dissatisfaction and disappointment with surgical outcomes.

The clinical significance of these nerve symptoms extends beyond physical discomfort. The negative impact of neuropathic pain and motor dysfunction on the physical and psychological well-being of patients is widely reported [7–10], with implications for quality of life (QoL), function, and healthcare costs. Studies show that patients with persistent neuropathic pain after KA experience significantly reduced QoL, with one study reporting health utility scores 34% lower than those in patients without such complications [11]. Wylde et al. found that patients with neuropathic symptoms after joint replacement report higher rates of anxiety (42% vs. 19%) and depression (38% vs. 12%) compared to those without such symptoms. [4] Furthermore, healthcare costs increase significantly when neuropathic symptoms persist after KA, with estimated additional costs of $7,000–$13,000 per patient annually due to increased healthcare utilization, medication use, and delayed return to normal activities [12]. These consequences highlight the importance of effectively managing peripheral nerve symptoms following KA.

Despite advancements in surgical techniques and perioperative management, a subset of KA recipients experience persistent peripheral nerve symptoms refractory to conventional treatments [13, 14]. Operative management of neuropathic pain and motor dysfunction after KA includes selective knee denervation, neurectomy of the infrapatellar branch of the saphenous nerve (IPBSN) [13–15], and common peroneal nerve (CPN) decompression. These techniques aim to alleviate neuropathic pain associated with symptomatic neuromas or compressive neuropathy [16, 17]. However, treatment strategies vary, and their efficacy is not consistent [5, 18, 19].

Given the common occurrence of neuropathic pain after KA and increasing interest of peripheral nerve surgeons in managing these symptoms, there remains an unmet need for a systematic approach to guide clinical decision-making. This study aims to evaluate the presentation, treatment, and outcomes of post-KA peripheral nerve symptoms and to propose an evidence-based surgical management algorithm based on our institutional experience. Our approach involves the integration of both traditional (nerve decompression) and emerging techniques (TMR and RPNI) for comprehensive management of this challenging problem. By identifying appropriate surgical interventions based on symptom characteristics and anatomic considerations, this work may significantly impact clinical practice, potentially improving QoL for the many patients who develop this problem.

## Methods

We performed a retrospective study and subsequent cross-sectional survey of adult patients who underwent peripheral nerve surgery for the treatment of peripheral nerve symptoms (defined as neuropathic pain and/or motor dysfunction) following KA (defined as partial or total KA) at a tertiary care center in the Northeastern United States. The cross-sectional survey assessed patient-reported outcome measures (PROMs) and QoL metrics of patients with a minimum follow-up of three months. All participants included in the survey provided written informed consent. This study was approved by the Institutional Review Board of Mass General Brigham (2020P003555). We adhered to the STROBE and SAMPL guidelines [20, 21].

### Patient population

**Fig. 1:**
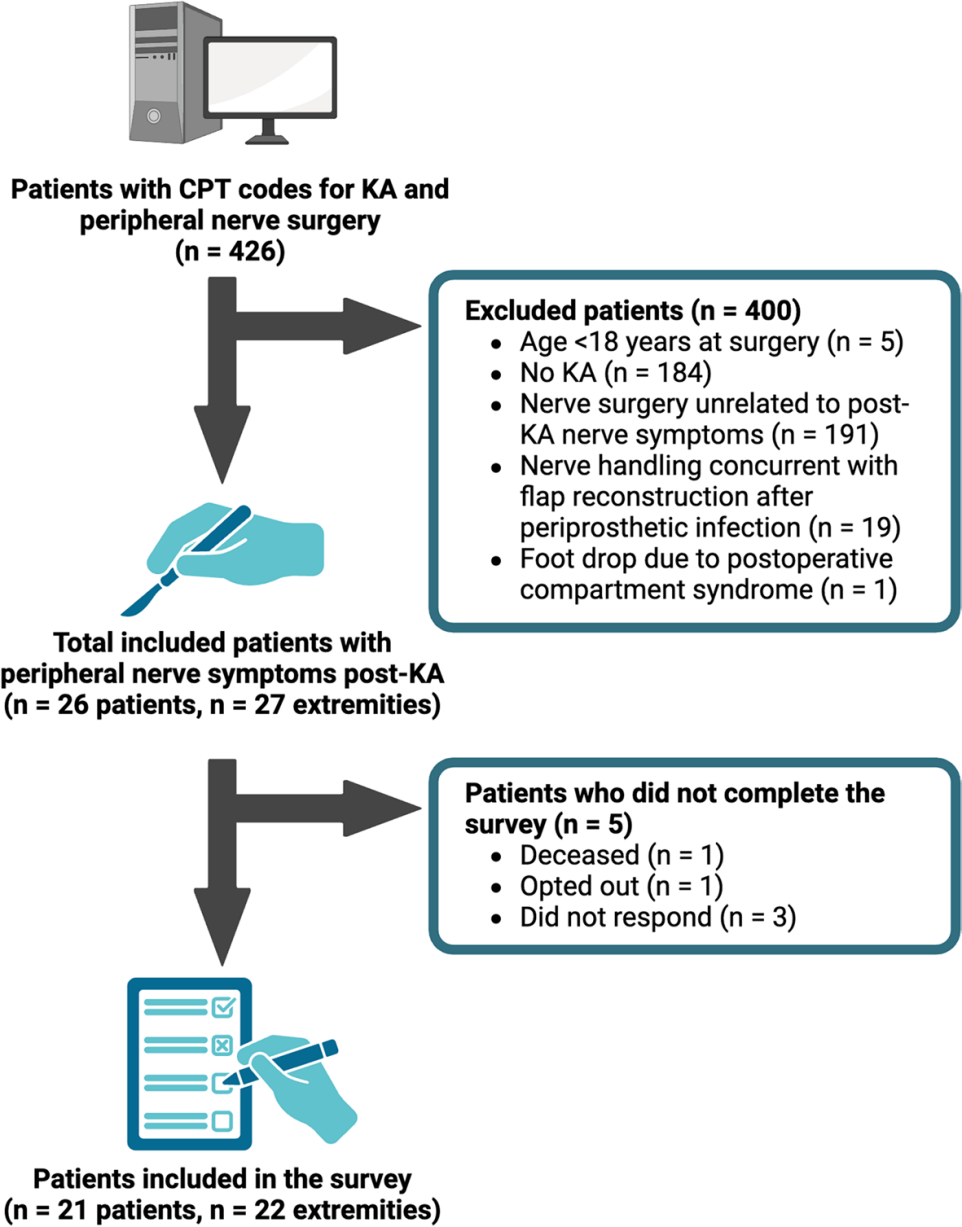
Flowchart of patient selection. A total of 426 patients were screened. Twenty-six patients with peripheral nerve symptoms after partial or total knee arthroplasty (KA) were included in the cohort study, and 21 patients completed the follow-up survey. CPT, Current Procedural Terminology; KA, knee arthroplasty

Patients were identified using International Classification of Diseases (ICD), Ninth Revision and Tenth Revision, and Current Procedural Terminology (CPT) codes for any peripheral nerve procedure between 2012 and 2024, if they did not undergo an amputation (Supplemental Digital Content 1). Initially, 3,058 patients were identified, of which 2,632 were excluded since they did not undergo KA in the ipsilateral lower extremity, according to their ICD or CPT codes. Of the 426 patients screened, patients were excluded if they did not undergo KA (*n* = 191) or if their nerve surgery was unrelated to the KA (*n* = 184). Patients were also excluded if nerve surgery was performed concurrently with flap harvest for soft tissue reconstruction after periprosthetic infection (*n* = 19), due to the potential confounding effects of inflammatory processes on nerve function and symptom presentation, which could obscure the assessment of primary nerve pathology. Similarly, patients were excluded if their peripheral nerve surgery was related to compartment syndrome (*n* = 1), as this represents a distinct pathophysiological entity requiring different management approaches. A total of 26 patients were included in the final analysis (Fig. 1), and 21 patients (80.8%) completed the cross-sectional survey.

### Patient selection

Clinical patient selection for peripheral nerve surgery followed a methodical and systematic approach. Initial evaluation included a comprehensive history focused on pain characteristics (quality, distribution, exacerbating/alleviating factors) and physical examination to identify specific nerve involvement. The timing of pain onset in relation to KA was carefully documented. For neuropathic pain, particularly on the medial aspect of the knee, diagnostic nerve blocks were performed under ultrasound guidance with 1–2 mL of 1% lidocaine to confirm the involved nerve territory, usually the saphenous nerve. A positive response to a block was defined as > 5 pts on the VAS pain score for the duration of the anesthetic effect. For patients with CPN-related symptoms, physical examination included testing of deep and superficial peroneal nerve function, assessment for Tinel's sign at the fibular neck, and testing of motor strength to dorsiflexion and eversion. Electrodiagnostic studies were ordered when motor weakness was present or suspected.

Criteria for consideration of peripheral nerve surgery included: 1) localized pain corresponding to specific peripheral nerve distributions, 2) positive response to diagnostic nerve block (if administered, and primarily for the saphenous nerve, 3) pain refractory to at least 3–6 months of conservative management (including medications, physical therapy, and interventional pain procedures), 4) absence of significant psychological comorbidities, and 5) realistic expectations about potential surgical outcomes. Patients with diffuse, non-anatomic pain patterns, negative response to diagnostic nerve blocks, or primary mechanical/nociceptive pain were generally not considered candidates for peripheral nerve surgery.

### Surgical technique

Neurectomy with TMR involves transection of the symptomatic nerve and coaptation of the proximal end to a motor nerve branch. This creates a physiologic end-organ target for regenerating axons, potentially preventing symptomatic neuroma formation by allowing organized nerve regeneration. RPNI similarly aims to provide a physiologic target for the severed nerve but involves placing the transected nerve into a small free muscle graft. For instance, in saphenous and IPBSN neurectomy, the nerve is approached through a medial longitudinal incision, identified proximal to the painful area, and transected. The proximal end is then either coapted to a motor branch of the vastus medialis muscle (TMR) or a free muscle graft is created to surround the nerve ending (RPNI).

CPN decompression involves an oblique longitudinal incision from the lateral aspect of the knee extending distally over the fibular head. The nerve is exposed in the peroneal tunnel, and all potential compression sites are released, including the intermuscular septae. This procedure aims to relieve mechanical compression or entrapment that may cause neuropathic pain or motor dysfunction.

### Data acquisition

All data were registered using Research Electronic Data Capture (REDCap 12.4.27, Vanderbilt University), an online and HIPAA-compliant data collection instrument [22]. Data collection methods included standardized chart review protocols with dual verification by two independent researchers to ensure accuracy and consistency. For subjective and qualitative data points, such as pain descriptions and physical examination findings, we employed a consensus approach when discrepancies occurred, with a third reviewer consulted when necessary.

Patient data on demographics, comorbidities, preoperative characteristics, and surgical characteristics were collected through chart review. Comorbidities included previously identified risk factors for neuropathic pain: alcoholism [23] smoking, diabetes (types 1 and 2), psychiatric comorbidities (depressive disorder, anxiety, posttraumatic stress disorder), hypothyroidism, peripheral vascular disease, chronic kidney disease, history of chronic pain, and complex regional pain syndrome (CRPS) [24, 25]. The use of opioids and neuromodulators both preoperatively and postoperatively was recorded. Peripheral nerve symptoms and surgery characteristics included previous lower extremity surgeries for post-KA peripheral nerve symptoms, indication for peripheral nerve surgery, use of a diagnostic peripheral nerve block, and (if applicable) the degree of CPN palsy [5]. Additionally, indications for KA, knee alignment deformity (> 10°) [26], KA laterality, time between KA and nerve surgery, treated nerves, surgical technique, and revision peripheral nerve surgery were recorded. Recovery from CPN palsy was evaluated based on the examination findings documented from the final surgical follow-up.

### Cross-sectional survey

A cross-sectional survey assessed PROMs, including pain, function, and QoL parameters. Participants completed standardized PROM questionnaires via telephone interview or electronically through the REDCap platform. The primary outcomes included the overall impression of meaningful change following surgery, measured by the Patient Global Impression of Change (PGIC, 7-point scale ranging from “very much worse” to “very much improved”) [27, 28], and health-related QoL, evaluated using the EuroQol European Quality of Life 5 Dimensions 5 Level (EQ-5D-5L) index, on a 0–1 scale [29, 30]. Secondary outcomes focused on pain levels measured using an 11-point (0, no pain—10, worst pain imaginable) numeric rating scale (NRS) [31], pain interference using Patient-Reported Outcomes Measurement Information System (PROMIS) short form (SF) 4a [32], and physical function, using PROMIS SF4a (PROMIS T-score mean and normal range is 50 (± 10) in the US general population) [33].

### Statistical analysis

Data analyses were performed with RStudio (version 2024.09.1 + 394, RStudio Team, PBC, Boston, MA) for R (version 4.4.1 (2024–06–14) MacOS, R Core Team, Vienna, Austria). The normality of continuous variables was assessed using Q-Q-plots and the Shapiro–Wilk test. Normally distributed variables were described as mean with ± SD, and non-normally distributed variables as median with interquartile range (IQR). Categorical variables are displayed as the raw number and percentage. No missing data was encountered, and no imputation was made for patients who did not complete the follow-up survey.

## Results

Of the 26 included patients, with a total of 27 treated lower extremities, the median age at the time of peripheral nerve surgery was 67.0 years (IQR: 58.0–71.8). Most patients were female (*n* = 16/26, 61.5%). Psychiatric comorbidities were present in 14 patients (53.8%), 13 patients (50.0%) were (former) smokers, and CRPS was diagnosed after KA but before peripheral nerve surgery in 2 patients (8.0%, *n* = 1 CRPS type I, *n* = 1 type II). See Table 1 for all demographic and comorbidity characteristics.

**Table 1:** Demographics and comorbidities

Variables	All patients, *n* = 26	Patients included in the survey, *n* = 21 (80.8%)
Demographics		
Age at time of surgery (years)	67.0 (58.0–71.8)	67.0 (56.0–72.0)
Body mass index (kg/m^2^)	29.0 (26.7–34.4)	28.5 (27.0–33.7)
Sex, female	16 (61.5)	14 (66.7)
Race, white	26 (100)	21 (100)
Hispanic ethnicity	1 (3.8)	1 (4.8)
Employment Status		
Disabled	2 (7.7)	1 (4.8)
Full-time	8 (30.8)	5 (23.8)
Part-time	1 (3.8)	0 (0)
Not employed	1 (3.8)	1 (4.8)
Retired	13 (50.0)	11 (52.4)
Insurance		
Medicare	16 (61.5)	14 (66.7)
Private	10 (38.5)	7 (33.3)
Follow-up^a^ (months)	*NA*	22.9 (12.9–50.2)
Comorbidities		
Alcoholism	2 (7.7)	2 (9.5)
Smoking	13 (50.0)	10 (47.6)
At time of surgery	2 (7.7)	2 (9.5)
Formerly	11 (42.3)	8 (38.1)
Opioid use, preoperatively	12 (46.2)	9 (42.9)
Opioid use, postoperatively	10 (38.5)	8 (38.1)
Neuromodulator use, preoperatively	13 (50.0)	9 (42.9)
Neuromodulator use, postoperatively	11 (42.3)	8 (38.1)
Diabetes	4 (15.4)	3 (14.3)
Hypothyroidism	5 (19.2)	5 (23.8)
Psychiatric comorbidity	14 (53.8)	10 (47.6)
Depressive disorder	8 (30.8)	6 (28.6)
Anxiety disorder	14 (53.8)	10 (47.6)
Post-traumatic stress disorder	1 (3.8)	1 (4.8)
Peripheral vascular disease	0 (0)	0 (0)
Chronic kidney disease	2 (7.7)	2 (9.5)
History of chronic pain	24 (92.3)	20 (95.2)
Complex regional pain syndrome	2 (7.7)	1 (4.8)

Numbers are presented as numbers (%) or medians (interquartile range)

^a^Follow-up survey was completed by *n* = 21/26 (80.8%) patients

The indication for KA was primary osteoarthritis in 23 surgeries (85.2%) and posttraumatic osteoarthritis in 4 surgeries (14.8%) (Table 2). Three extremities (11.1%) had a valgus and 4 (14.8%) had a varus deformity. Total KA was performed in 24 surgeries (88.9%) and partial KA in 3 surgeries (11.1%). In five extremities (18.5%), more than one knee joint arthroplasty surgery was conducted prior to presenting for peripheral nerve surgery at our clinic. Indications for peripheral nerve surgery included chronic pain (*n* = 24/27, 89%), CPN palsy causing foot drop (*n* = 1/27, 4%), or both (*n* = 2/27, 7%). CPN palsy was classified as incomplete in all three patients presenting with foot drop.

**Table 2:** Preoperative characteristics

Variables	All lower extremities^a^, *n* = 27	Extremities of patients included in the survey, *n* = 22 (81.5%)
Affected lower extremity		
Left	8 (30.8)	5 (23.8)
Right	17 (65.4)	15 (71.4)
Bilateral^a^	1 (3.8)	1 (4.8)
Indications for knee arthroplasty		
Osteoarthritis	23 (85.2)	19 (86.4)
Posttraumatic osteoarthritis	4 (14.8)	3 (13.6)
Alignment deformity		
Valgus	3 (11.1)	2 (9.1)
Varus	4 (14.8)	3 (13.6)
Knee arthroplasty type		
Total knee arthroplasty	24 (88.9)	19 (86.4)
Partial knee arthroplasty	3 (11.1)	3 (13.6)
Total number of knee arthroplasties in the treated leg		
1	22 (81.5)	18 (81.8)
2	4 (14.8)	3 (13.6)
3	1 (3.7)	1 (4.5)
Indications for nerve surgery		
Chronic pain	24 (88.9)	20 (90.9)
CPN palsy (foot drop)	1 (3.7)	0 (0.0)
Combination	2 (7.4)	2 (9.1)
Cause of nerve injury		
Iatrogenic	26 (96.3)	21 (95.5)
Trauma	1 (3.7)	1 (4.5)
Classification of CPN palsy at diagnosis		
Incomplete palsy	3 (100.0)	2 (9.1)
Complete palsy	0 (0.0)	0 (0.0)
Diagnostic peripheral nerve block	16 (59.3)	14 (63.6)
Previous peripheral nerve surgery in the same neurotome^b^	5 (18.5)	5 (22.7)
*No. surgeries:* 1	2 (7.4)	2 (9.1)
2	1 (3.7)	1 (4.5)
3	1 (3.7)	1 (4.5)
5	1 (3.7)	1 (4.5)
Time between arthroplasty and nerve surgery (months)	29.5 (12.5–71.0)	39.9 (13.1–73.3)

*CPN* common peroneal nerve

^a^*n* = 27 lower extremities treated in *n* = 26 patients due to one case of sequential bilateral treatment

^b^Procedures included saphenous (6) and IPBSN (1) neurectomy, IPBSN allograft reconstruction (2), CPN decompression (1), and spinal cord stimulator trial (1) and definitive implantation (1)

**Fig. 2:**
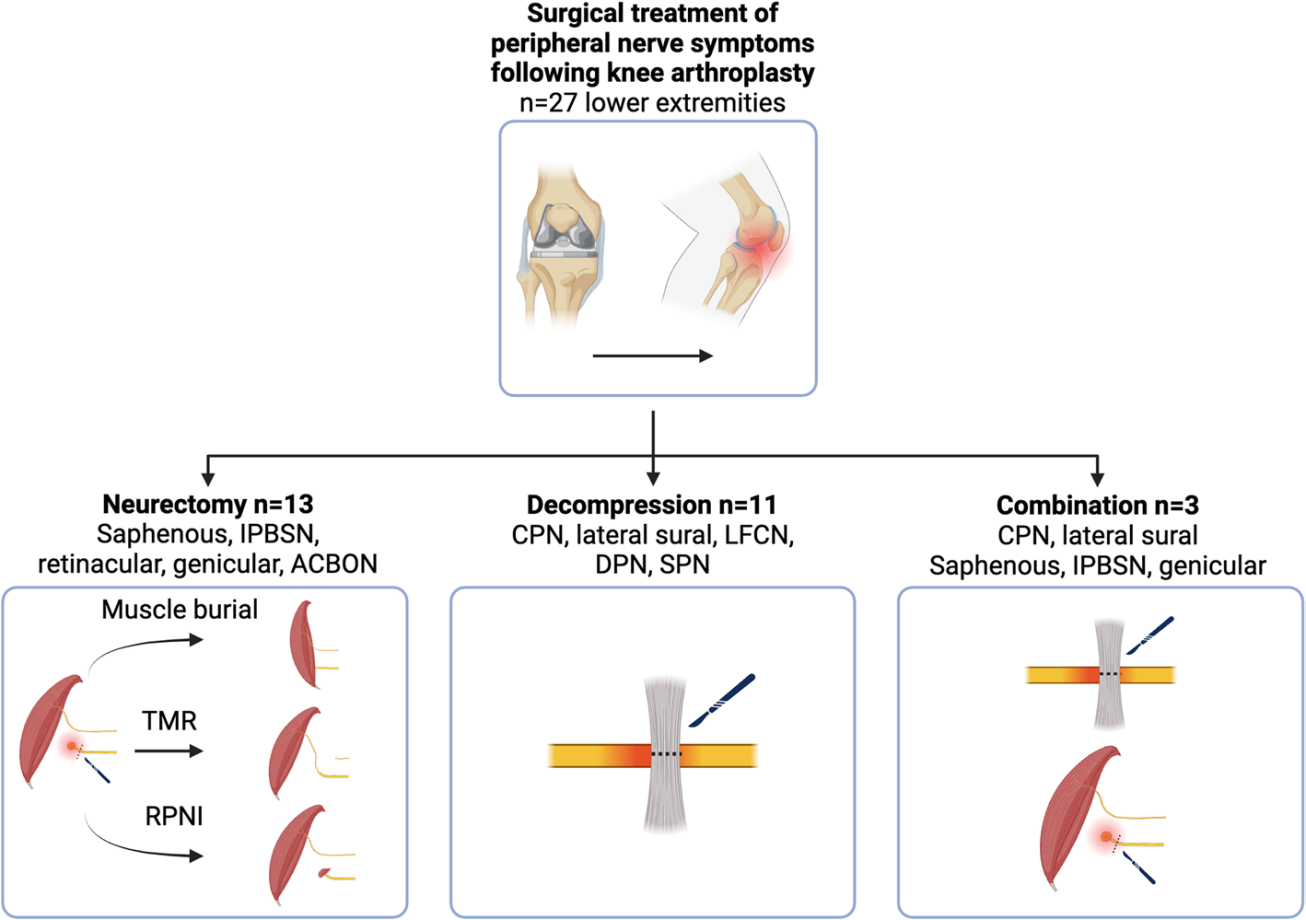
Surgical management of peripheral nerve symptoms following knee arthroplasty. Of the 27 treated extremities, neurectomy followed by muscle burial, TMR, or PRNI was conducted in *n* = 13, nerve decompression in *n* = 11, and a combination of these techniques in *n* = 3 extremities. ACBON, anterior cutaneous branch of the obturator nerve; CPN, common peroneal nerve; DPN, deep peroneal nerve; IPBSN, infrapatellar branch of the saphenous nerve; LFCN, lateral femoral cutaneous nerve; SPN, superficial peroneal nerve; RPNI, regenerative peripheral nerve interface; TMR, targeted muscle reinnervation

In 5 lower extremities (18.5%), previous peripheral nerve surgery was conducted in the same nerve distribution prior to referral to our clinic and included saphenous and IPBSN neurectomy, IPBSN allograft reconstruction, CPN decompression, and a spinal cord stimulator. A diagnostic nerve block was administered in 16 (66.7%) of the extremities affected by neuropathic pain, leading to pain relief in 13 cases (81.3%). The median interval between KA and peripheral nerve surgery at our clinic was 29.5 months (IQR: 12.5–72.0). Surgical procedures included neurectomy of the saphenous nerve or IPBSN with active (TMR or RPNI) or passive (excision with or without implantation) nerve management [34] (*n* = 13/27, 48.1%), CPN decompression (*n* = 11/27, 40.7%) and surgical combinations thereof (*n* = 3/27, 11.1%) (Fig. 2). In the neurectomy group, the primary treatment was saphenous nerve neurectomy (mostly with TMR) in nine extremities (69.2%) and IPBSN neurectomy (with implantation into muscle, RPNI, or allograft relocation nerve grafting [35, 36]) in four extremities (30.8%). Neurectomy of additional nerves during the same surgery was performed in nine extremities at the operating surgeon’s discretion (69.2%), including the retinacular, genicular, and anterior cutaneous branch of the obturator nerve (ACBON). In the decompression group, lateral sural nerve decompression was conducted in addition to CPN decompression in six extremities (54.5%). In three cases, other nerves were decompressed during the same surgery in addition to the CPN: the lateral femoral cutaneous nerve (LFCN), the superficial peroneal nerve (SPN), and the deep peroneal nerve (DPN). The combined treatment group included three cases of CPN and lateral sural nerve decompression in combination with neurectomy of the: 1) saphenous nerve, 2) saphenous and medial genicular nerves, and 3) IPBSN. Details on the surgical technique per nerve and the combination of nerves treated during the same surgery can be found in Supplemental Digital Content 2 and 3. No revision surgeries for continued symptoms were conducted. Among the three patients with incomplete CPN palsy, 3/3 (100%) had recovery with improved motor function after nerve decompression.

### Patient-reported outcomes

**Fig. 3:**
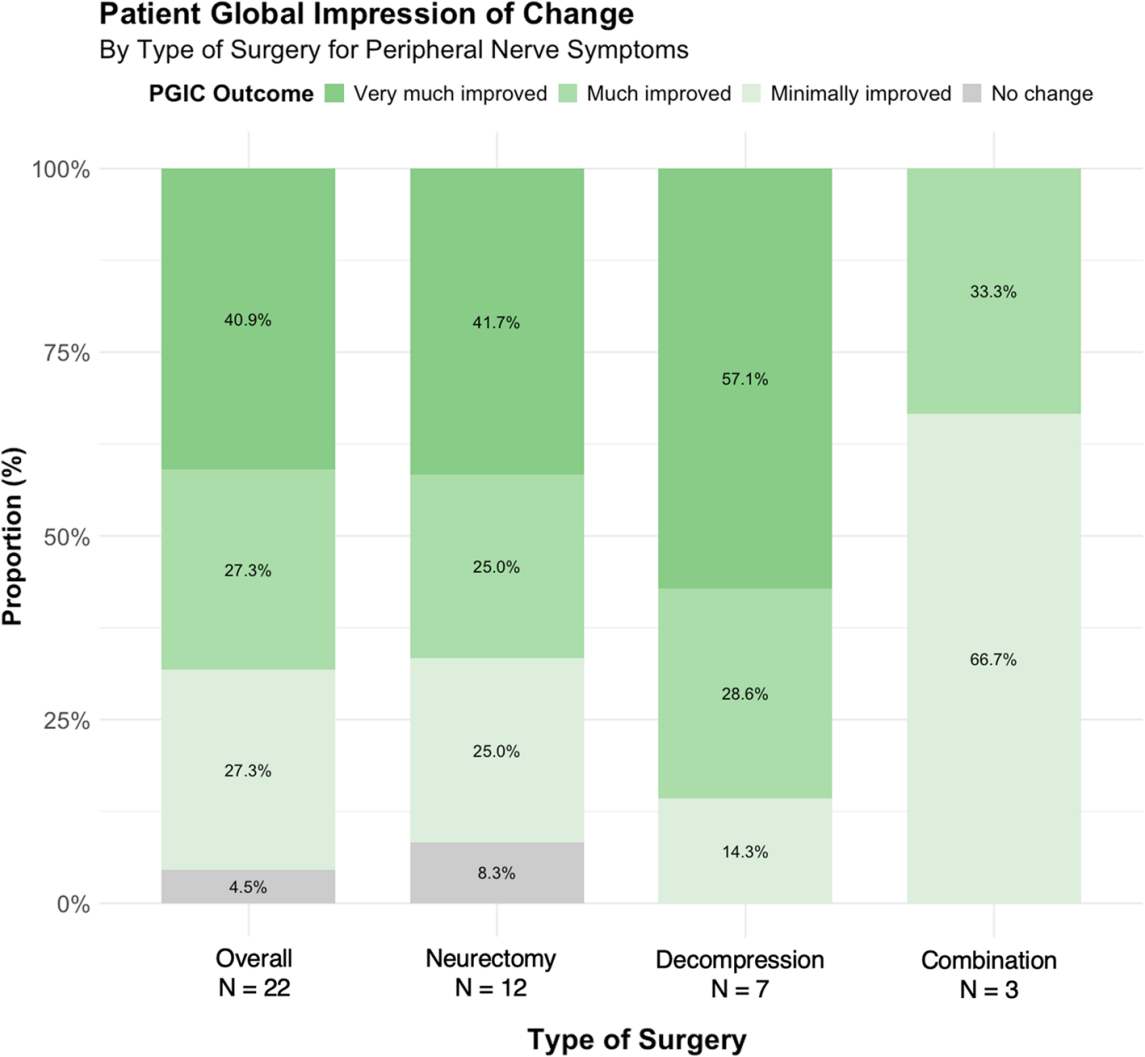
Patient Global Impression of Change by Type of Surgery for Peripheral Nerve Symptoms. Reported using the PGIC questionnaire. Overall (*n* = 22): 78.5% improvement (45.0% very much improved, 33.3% much improved, 21.4% minimally improved), 13.5% no change, 8.0% worsening (4.0% minimally worse, 4.0% much worse). Neurectomy (*n* = 12): 75.0% improvement (41.7% very much improved, 33.3% much improved), 16.7% no change, 8.3% worsening. Decompression (*n* = 7): 85.7% improvement (57.1% very much improved, 28.6% much improved), 14.3% no change. Combination (*n* = 3): 66.7% improvement (33.3% very much improved, 33.3% much improved), 33.3% no change. PGIC, Patient Global Impression of Change

Twenty-one of the 26 (80.8%) patients completed the cross-sectional follow-up survey (*n* = 22/27 extremities included, 81.5%). Demographics and surgical characteristics of survey respondents were comparable to the overall cohort (Tables 1 and 2). The median follow-up time was 1.9 years (IQR: 1.1–4.2) after surgery. In twenty-one surgical cases (95.5%), improvement of peripheral nerve symptoms after peripheral nerve surgery was reported, as assessed by the PGIC: minimally (*n* = 6/22, 27.3%), much (*n* = 6/22, 27.3%), or very much improved (*n* = 9/22, 40.9%). This included one of the patients with CRPS (type I) who underwent IPBSN and genicular nerve neurectomy with transposition into muscle and reported minimal improvement. No change was reported for one extremity (4.5%) by a patient in the neurectomy group who underwent knee denervation with submuscular burial of nerve ends (Table 3, Fig. 3). The mean EQ-5D-5L index for health-related QoL was 0.861 (± 0.105), compared to the US general population mean of 0.851 (± 0.205) [30]. In the neurectomy group, this index was 0.840 (± 0.079), in the decompression group, 0.944 (± 0.096), and in the combined treatment group, 0.751 (± 0.090) (Table 3).

**Table 3:** Patient-reported outcome measures after nerve surgery for post-knee arthroplasty peripheral nerve symptoms

Patient reported outcome measures				
Patient global impression of change				
	Overall *n* = 22^a^	Neurectomy *n* = 12	Decompression *n* = 7	Combination *n* = 3
Very much improved	9 (40.9)	5 (41.7)	4 (57.1)	0 (0.0)
Much improved	6 (27.3)	3 (25.0)	2 (28.6)	1 (33.3)
Minimally improved	6 (27.3)	3 (25.0)	1 (14.3)	2 (66.7)
No change	1 (4.5)	1 (8.3)	0 (0.0)	0 (0.0)
Minimally worse	0 (0.0)	0 (0.0)	0 (0.0)	0 (0.0)
Much worse	0 (0.0)	0 (0.0)	0 (0.0)	0 (0.0)
Very much worse	0 (0.0)	0 (0.0)	0 (0.0)	0 (0.0)
Pain, Physical Function & Quality of Life				
	Overall *n* = 22^a^	Neurectomy *n* = 12	Decompression *n* = 7	Combination *n* = 3
Pain score (NRS/VAS)	3.9 (± 3.2)	5 (± 3.2)	0.9 (± 1.4)	5.6 (± 2.0)
Pain Interference	59.5 (± 9.4)	62.7 (± 6.4)	51.9 (± 10.9)	64.2 (± 6.4)
Physical Function	41.3 (± 8.4)	38.5 (± 4.0)	48.8 (± 10.4)	34.8 (± 4.1)
Quality of Life^b^	0.861 (± 0.105)	0.840 (± 0.079)	0.944 (± 0.096)	0.751 (± 0.090)

*NRS* numeric rating scale, *VAS* visual analog scale [36]

^a^Included 21/26 (80.7%) patients reporting on 22/27 (81.4%) treated lower extremities at a median follow-up of 22.9 (IQR: 12.9–50.2) months postoperatively

^b^United States EQ-5D-5L population norm 0.851 (± 0.205) [35]

The secondary outcomes pain level (NRS) and pain interference (PROMIS T-score) had a mean of respectively 3.9 (± 3.2) and 59.5 (± 9.4) overall, 5 (± 3.2) and 62.7 (± 6.4) in the neurectomy group, 0.9 (± 1.4) and 51.9 (± 10.9) in the decompression group, and 5.6 (± 2.0) and 64.2 (± 6.4) in the combined treatment group. Physical function exhibited a mean PROMIS T-score of 41.3 (± 8.4) in the overall cohort, 38.5 (± 4.0) in the neurectomy group, 48.8 (± 10.4) in the decompression group, and 34.8 (± 4.1) in the combined treatment group (Table 3).

## Discussion

In this retrospective study, we found that patients with persistent neuropathic pain, numbness, or weakness after KA surgery benefited from peripheral nerve surgery. At a median follow-up of approximately two years, 95.5% of patients reported improvement of peripheral nerve symptoms and achieved a QoL comparable to the US general population.

As KA is common [1], recognition of treatment options for peripheral nerve symptoms after KA is of substantial clinical relevance. Differentiating between nociceptive knee pain and neuropathic pain can be challenging. Neuropathic pain often presents as burning, shooting, or electrical-type pain, sometimes with numbness and/or paresthesias as well as allodynia, hyperalgesia, and a Tinel sign. In contrast, nociceptive knee pain can be related to movement or mechanical stress.

**Fig. 4:**
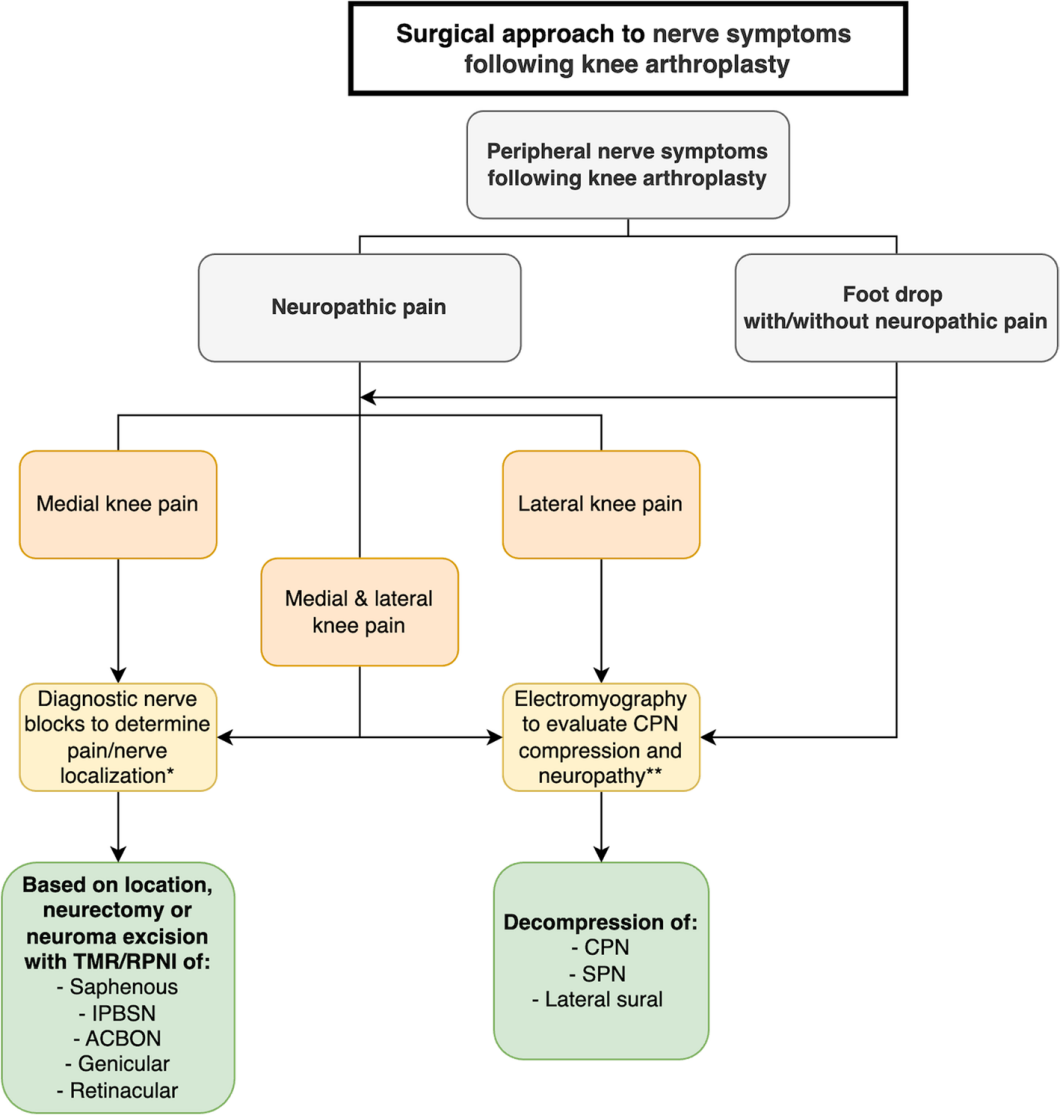
Surgical treatment algorithm flowchart for patients presenting with neuropathic symptoms after knee arthroplasty in a peripheral nerve surgery practice. *When a diagnostic peripheral nerve block (mixture of lidocaine and bupivacaine) leads to clinically substantial pain relief (ideally > 50%), it suggests a focused peripheral nerve etiology, and surgical intervention for the suspected causative nerve is considered. **CPN motor deficits are diagnosed by clinical examination and EMG. ACBON, anterior cutaneous branch of the obturator nerve; CPN, common peroneal nerve; IPBSN, infrapatellar branch of the saphenous nerve; SPN, superficial peroneal nerve; RPNI, regenerative peripheral nerve interface; TMR, targeted muscle reinnervation

Often, non-surgical pain management interventions for patients with neuropathic pain following KA are initially employed. Ultrasound-guided injections with local anesthesia and/or corticosteroids, as well as radiofrequency ablation of the IPBSN and genicular nerves, have been described. These non-surgical techniques may offer a limited durability of pain relief [13, 37, 38]. It is reasonable first to consider non-surgical options for patients with ongoing neuropathic symptoms following KA, but peripheral nerve surgery has an important role and should be considered if symptoms persist or evolve. We propose a surgical treatment algorithm for post-KA peripheral nerve symptoms based on our findings, experience, and the literature (Fig. 4).

Consideration of the anatomic location of pain is critical to determine the most appropriate treatment strategy. For patients experiencing anterolateral leg symptoms with dorsiflexion and eversion weakness or complete foot drop (i.e., symptoms related to the CPN), decompression of the CPN, SPN, and/or lateral sural nerves may offer benefit. Electromyography (EMG) can be a helpful diagnostic adjunct to evaluate CPN neuropathy in these patients [39]. In our experience, EMG does not always demonstrate a concrete neurogenic etiology, even if successful peripheral nerve surgery is later employed. If acute mechanical nerve entrapment is the likely etiology, such as in CPN palsy, early surgical decompression may be considered [40, 41]. While conservative management of CPN palsy is described, complete recovery has been reported to take up to two years or more [42, 43]. Some studies advocate for nerve decompression if there is no clinical improvement of CPN palsy within three months [44, 45]. In one case series, full recovery was seen at an average follow-up of three months in all patients who underwent acute CPN release for palsy at a mean of 18 days after KA, suggesting a potential benefit of early nerve surgery [41, 46, 47].

Surgical decompression has been shown to treat CPN dysfunction [48]. In our study, patients who underwent decompression reported a mean NRS score of 0.9. They achieved Pain Interference, Physical Function, and QoL scores similar to those of the general US population, highlighting the favorable outcomes of this approach. Additionally, one case report showed pain relief and improved function after CPN decompression in a patient presenting with CPN dysfunction ten years post-KA [49], further supporting our findings. In our study, all patients undergoing decompression had clinical improvement in their peripheral nerve symptoms. The significant reduction in pain scores observed in the decompression group compared to the neurectomy group likely reflects differential nerve pathologies, as compressive neuropathy is frequently treated successfully with surgery. As a result, we believe that CPN-related symptoms are a common and often overlooked cause of neuropathic symptoms following KA and are likely to improve with intervention.

In patients presenting with anterior or medial knee pain after KA, we recommend determining the precise anatomic location of the involved/painful nerve utilizing diagnostic nerve blocks [50]. We typically begin with either a genicular nerve block or a saphenous nerve block in the distal thigh to determine if the pain is transiently improved. If surgery is considered, we often perform neurectomy with active surgical management of the saphenous nerve [34], consisting of TMR/RPNI of the main saphenous nerve or the IPBSN. Dellon's work demonstrated that selective denervation could relieve pain in up to 86.0% of patients with intractable knee pain after total knee arthroplasty [40, 51]. Current literature on surgical management of neuropathic pain post-KA primarily describes IPBSN neurectomy and partial knee denervation with passive nerve management [34]. typically consisting of excision and nerve implantation into muscle or bone [15, 18, 52, 53]. With only one patient (8.3%) of the neurectomy group reporting no improvement of peripheral nerve symptoms in our study, results are comparable to some recent studies with passive nerve management reporting 4–8% of patients with no improvement postoperatively [15, 52]. However, while Shi et al. [52] reported reoperations for recurrent pain in 6% of patients undergoing knee denervation, we noted no revision surgeries in our cohort.

Careful patient selection remains paramount. The anatomic location of pain, combined with selective EMG and diagnostic nerve blocks, should guide surgical decision-making to identify which patients are most likely to benefit from nerve surgery [40, 51]. Our clinical approach to patient selection is systematic. First, the timing of referral varies widely—patients with motor symptoms typically present within weeks to months after KA. In contrast, those with neuropathic pain are often referred months to years later after having explored various conservative and non-operative treatments. Prolonged time between pain and treatment negatively impacts the outcome of surgery. We recommend diagnostic peripheral nerve blocks under ultrasound guidance to assess the presence of peripheral-mediated pain and to evaluate pain relief [50]. Surgical intervention is considered when there is a focused peripheral nerve etiology, as well as the location of the symptoms. Surgical considerations include the application of passive (simple neurectomy or implantation into muscle) versus active (TMR and RPNI) management of nerve endings after neurectomy [34].

The observed differences in efficacy between surgical approaches in our study likely reflect the distinct underlying pathophysiologies being addressed. Nerve decompression procedures demonstrated the highest efficacy (mean NRS pain score 0.9 ± 1.4) compared to neurectomy (5.0 ± 3.2) and combined approaches (5.6 ± 2.0). This pattern may be attributed to the nature of compressive neuropathies, which often respond more completely to surgical intervention. In contrast, neurectomy approaches address neuropathic pain from terminal nerve branches or neuromas, and result in Wallerian degeneration as well as axonal regeneration from the proximal nerve stump, which is a more complex physiologic process than recovery from decompression alone. The combined approach group showed the lowest improvement in physical function (PROMIS T-score 34.8 ± 4.1 vs. 48.8 ± 10.4 for decompression), possibly reflecting the more complex pathology in these patients requiring both neurectomy and decompression procedures.

Our findings build upon previous research in several important ways. While prior studies have demonstrated the efficacy of IPBSN neurectomy with passive management of nerve endings [18] and selective knee denervation [52], our study introduces active nerve management strategies (TMR and RPNI). Shi et al. [52] reported a 6% revision rate for recurrent pain after knee denervation, compared to no revisions in our cohort. For motor symptoms, Park et al. [42] documented variable recovery with conservative management (50% full recovery, 29% partial, 21% none), while our finding of 100% improvement with surgical decompression aligns with Johnson et al., [41] who reported complete recovery following acute CPN release. Our study uniquely combines both pain and motor symptom management in a single cohort, whereas most prior studies address either neuropathic pain [15] or CPN palsy [5] separately. We believe that this allows us to propose a comprehensive treatment algorithm.

The clinical significance of our findings extends beyond statistical improvements in outcome measures. For patients experiencing persistent neuropathic symptoms after KA—a population often subjected to multiple unsuccessful interventions—our results demonstrate that targeted peripheral nerve procedures can significantly improve QoL and function. The demonstrated efficacy of CPN decompression for lateral knee pain and foot drop suggests that this relatively straightforward procedure should be considered earlier in the treatment algorithm, potentially before more invasive interventions such as revision arthroplasty or long-term opioid therapy are initiated. Similarly, the success of saphenous nerve neurectomy with active management for medial knee pain provides surgeons with a viable option for patients previously considered to have pain of uncertain etiology or presumed to have failed arthroplasty. Implementation of our proposed algorithm in clinical practice may streamline the diagnostic process, reduce unnecessary interventions, and accelerate appropriate referrals to peripheral nerve surgeons, ultimately improving patient care efficiency and outcomes.

This study has limitations. The small sample size in our study limits the statistical power and our ability to conduct subgroup analyses comparing different surgical approaches. The lack of pre-nerve surgery outcome data prevents quantification of the magnitude of improvement following surgery, although the PGIC was specifically employed to capture patients'subjective assessment of meaningful change, and EQ-5D-5L was selected as a holistically relevant outcome. The PGIC, while validated and widely used, relies on subjective patient reporting and may be influenced by recall bias. Furthermore, our follow-up completion rate of 80.8%, while sufficient, raises the possibility of selection bias, as patients with poorer outcomes might be less likely to complete follow-up surveys.

Future studies should prospectively collect standardized preoperative and postoperative outcome measures with larger cohorts to validate our findings. Additionally, incorporating validated neuropathic pain (e.g., DN4, PainDETECT) and KA-specific functional outcome (e.g., Oxford Knee Score) assessment tools, and conducting longer follow-up would strengthen future investigations and enable more robust statistical analyses. Although our observations provide valuable insights into the intermediate-term outcomes of peripheral nerve surgery after KA, with sustained improvement in 95.5% of patients at a median follow-up of about two years, longer-term data are required to further elucidate the durability of these interventions. Potential late complications, such as symptomatic neuroma recurrence at different sites following neurectomy or recurrent compression after initial decompression, may emerge over time.

## Conclusions

In patients with neuropathic pain, numbness, or weakness after KA, peripheral nerve surgery may be beneficial, with 95% of patients reporting improvement at approximately two years’ follow-up. We recommend CPN and/or SPN and lateral sural nerve decompression for lateral knee pain and/or foot drop, active nerve management using TMR or RPNI of the saphenous nerve for medial knee pain, and combinations for both. The proposed algorithm not only optimizes management of these patients but may significantly improve QoL, reduce healthcare utilization associated with persistent pain, and enable more complete functional recovery. By providing a structured approach to a challenging clinical problem, these findings have the potential to transform care pathways for the many patients who develop nerve symptoms following the multitude of KA procedures performed annually in the United States and abroad.

## Supplementary Information

Supplementary Material 1.

Supplementary Material 2.

Supplementary Material 3.

## Data Availability

The dataset generated and analyzed during the current study is not publicly available due to patient privacy concerns, but is available from the corresponding author on reasonable request.

## Abbreviations

KA: Knee Arthroplasty
TMR: Targeted Muscle Reinnervation
RPNI: Regenerative Peripheral Nerve Interface
CPN: Common Peroneal Nerve
IPBSN: Infrapatellar Branch of the Saphenous Nerve
CRPS: Complex Regional Pain Syndrome
QoL: Quality of Life
PGIC: Patient Global Impression of Change
EQ-5D-5L: EuroQol 5 Dimension 5 Level
NRS: Numeric Rating Scale
PROMIS: Patient-Reported Outcomes Measurement Information System
EMG: Electromyography
SPN: Superficial Peroneal Nerve
DPN: Deep Peroneal Nerve
LFCN: Lateral Femoral Cutaneous Nerve
ACBON: Anterior Cutaneous Branch of the Obturator Nerve
