# Supplementary material for: Surgical management of peripheral nerve symptoms following knee arthroplasty
Source: Arthroplasty. 2025 Jun 6;7:27. doi: 10.1186/s42836-025-00315-0 (PMC12142898; doi:10.1186/s42836-025-00315-0)
Supplement: Supplementary file 1 — Supplementary Material 1. [file 42836_2025_315_MOESM1_ESM.docx]

**Supplemental Digital Content 1.** Codes used for identification of patients

Patient identification request at the institutional Research Patient Data Registry:

**NOT one of the following codes**

| **Description of lower extremity amputation** | **Code type** | **Code** |
| --- | --- | --- |
| Amputation of lower limb | ICD9P | 84.1 |
| Lower limb amputation, not otherwise specified | ICD9P | 84.1 |
| Amputation through foot | ICD9P | 84.12 |
| Disarticulation of ankle | ICD9P | 84.13 |
| Amputation of ankle through malleoli of tibia and fibula | ICD9P | 84.14 |
| Other amputation below knee | ICD9P | 84.15 |
| Disarticulation of knee | ICD9P | 84.16 |
| Amputation above knee | ICD9P | 84.17 |
| Disarticulation of hip | ICD9P | 84.18 |
| Abdominopelvic amputation | ICD9P | 84.19 |
| Hindquarter, Right | ICD10P | 0Y62 |
| Hindquarter, Left | ICD10P | 0Y63 |
| Hindquarter, Bilateral | ICD10P | 0Y64 |
| Femoral Region, Right | ICD10P | 0Y67 |
| Femoral Region, Left | ICD10P | 0Y68 |
| Upper Leg, Right | ICD10P | 0Y6C |
| Upper Leg, Left | ICD10P | 0Y6D |
| Knee Region, Right | ICD10P | 0Y6F |
| Knee Region, Left | ICD10P | 0Y6G |
| Lower Leg, Right | ICD10P | 0Y6H |
| Lower Leg, Left | ICD10P | 0Y6J |
| Foot, Right | ICD10P | 0Y6M |
| Foot, Left | ICD10P | 0Y6N |
| Amputation, thigh, through femur, any level | CPT | 27590 |
| Amputation, thigh, through femur, any level; immediate fitting technique including first cast | CPT | 27591 |
| Amputation, thigh, through femur, any level; open, circular (guillotine) | CPT | 27592 |
| Amputation, thigh, through femur, any level; secondary closure or scar revision | CPT | 27594 |
| Disarticulation at knee | CPT | 27598 |
| Amputation, leg, through tibia and fibula | CPT | 27880 |
| Amputation, leg, through tibia and fibula; with immediate fitting technique including application of first cast | CPT | 27881 |
| Amputation, leg, through tibia and fibula; open, circular (guillotine) | CPT | 27882 |
| Amputation, leg, through tibia and fibula; re-amputation | CPT | 27886 |
| Amputation, ankle, through malleoli of tibia and fibula (eg, Syme, Pirogoﬀ type procedures), with plastic closure and resection of nerves | CPT | 27888 |
| Ankle disarticulation | CPT | 27889 |
| Amputation, foot; midtarsal (eg, Chopart type procedure) | CPT | 28800 |
| Amputation, foot; transmetatarsal | CPT | 28805 |

**AND either of the following codes**

| **Description of nerve procedure** | **Code type** | **Code** |
| --- | --- | --- |
| Neuroplasty; nerve of hand or foot | CPT | 64704 |
| Neuroplasty, major peripheral nerve, arm or leg; other than specified | CPT | 64708 |
| Neuroplasty, major peripheral nerve, arm or leg; sciatic nerve | CPT | 64712 |
| Internal neurolysis, requiring use of operating microscope | CPT | 64727 |
| Grafting of autologous soft tissue, other, harvested by direct excision | CPT | 15769 |
| Implantation of nerve end into bone or muscle | CPT | 64787 |
| Excision of neuroma; major peripheral nerve, except sciatic | CPT | 64784 |
| Excision of neuroma; hand or foot, except digital nerve | CPT | 64782 |
| Excision of neuroma; cutaneous nerve, surgically identifiable | CPT | 64774 |
| Decompression; unspecified nerve(s) | CPT | 64722 |
| Transection or avulsion of other spinal nerve, extradural | CPT | 64772 |
| Unlisted procedure, nervous system | CPT | 64999 |

CPT= Current Procedural Terminology, ICD=International Classification of Diseases, ICD9P= ICD 9th revision procedure code, ICD9D= ICD 9th revision diagnosis code, ICD10P= ICD 10th revision procedure code, ICD10D= ICD 10th revision diagnosis code
