# Supplementary material for: Surgical management of peripheral nerve symptoms following knee arthroplasty
Source: Arthroplasty. 2025 Jun 6;7:27. doi: 10.1186/s42836-025-00315-0 (PMC12142898; doi:10.1186/s42836-025-00315-0)
Supplement: Supplementary file 2 — Supplementary Material 2. [file 42836_2025_315_MOESM2_ESM.docx]

**Supplemental Digital Content 2.** Surgical description and frequencies per treated nerve (n=54)

| **Treated nerves** | **Frequency** | **Surgical descriptions per nerve** | **Frequency** |
| --- | --- | --- | --- |
| **CPN** | 14 | Decompression | 12 |
|  |  | Neurolysis | 1 |
|  |  | Neurolysis and decompression | 1 |
| **Saphenous main branch** | 11 | TMR | 10 |
|  |  | Neurectomy | 1 |
| **Lateral sural** | 9 | Decompression | 9 |
| **IPBSN** | 5 | Neuroma excision and allograft reconstruction with intramuscular burial | 1 |
|  |  | Neurectomy and intramuscular burial | 3 |
|  |  | Neurolysis, neuroma excision, and RPNI | 1 |
| **Lateral retinacular** | 4 | Neurectomy with muscle burial | 3 |
|  |  | Neurolysis, neuroma excision, and RPNI | 1 |
| **ACBON** | 3 | RPNI  TMR | 2  1 |
| **Medial retinacular** | 3 | Neurectomy with muscle burial | 3 |
| **Medial genicular** | 2 | Neurectomy with muscle burial | 2 |
| **LFCN** | 1 | Decompression | 1 |
| **DPN** | 1 | Decompression | 1 |
| **SPN** | 1 | Decompression | 1 |

Abbreviations: ACBON, anterior cutaneous branch of the obturator nerve; CPN, common peroneal nerve; DPN, deep peroneal nerve; IPBSN, infrapatellar branch of the saphenous nerve; SPN, superficial peroneal nerve; RPNI, regenerative peripheral nerve interface; TMR, targeted muscle reinnervation.
