# Supplementary material for: Surgical management of peripheral nerve symptoms following knee arthroplasty
Source: Arthroplasty. 2025 Jun 6;7:27. doi: 10.1186/s42836-025-00315-0 (PMC12142898; doi:10.1186/s42836-025-00315-0)
Supplement: Supplementary file 3 — Supplementary Material 3. [file 42836_2025_315_MOESM3_ESM.docx]

**Supplemental Digital Content 3.** Frequencies and combinations of all treated nerves (n=54)

| **Nerves treated during the same surgery in patients** | | | | |
| --- | --- | --- | --- | --- |
| **Nerve 1** | **Nerve 2** | **Nerve 3** | **Nerve 4** | **Frequency** |
| **Saphenous main branch** |  |  |  | 3 |
| **Saphenous main branch** | ACBON |  |  | 3 |
| **Saphenous main branch** | Medial retinacular |  |  | 1 |
| **Saphenous main branch** | Lateral retinacular |  |  | 1 |
| **Saphenous main branch** | Lateral retinacular | Medial retinacular |  | 1 |
| **IPBSN** |  |  |  | 1 |
| **IPBSN** | Medial genicular |  |  | 1 |
| **IPBSN** | Lateral retinacular |  |  | 1 |
| **IPBSN** | Lateral retinacular | Medial retinacular |  | 1 |
| **CPN** |  |  |  | 3 |
| **CPN** | Lateral sural |  |  | 6 |
| **CPN** | LFCN |  |  | 1 |
| **CPN** | DPN | SPN |  | 1 |
| **CPN** | Lateral sural | IPBSN |  | 1* |
| **CPN** | Lateral sural | Saphenous main branch |  | 1* |
| **CPN** | Lateral sural | Saphenous main branch | medial genicular | 1* |

*These three extremities are part of the combined (neurectomy + decompression) treatment groups. Abbreviations: ACBON, anterior cutaneous branch of the obturator nerve; CPN, common peroneal nerve; DPN, deep peroneal nerve; IPBSN, infrapatellar branch of the saphenous nerve; SPN, superficial peroneal nerve; RPNI, regenerative peripheral nerve interface; TMR, targeted muscle reinnervation.
